# Pathogenesis of Ventricular Arrhythmias and Its Effect on Long-Term Prognosis in Patients With Takotsubo Cardiomyopathy

**DOI:** 10.7759/cureus.11171

**Published:** 2020-10-26

**Authors:** Julio A Pena Escobar, Myat Aung, Saba Amin, Azouba Gulraiz, Fenil R Gandhi, Bilal Haider Malik

**Affiliations:** 1 Internal Medicine, California Institute of Behavioral Neurosciences & Psychology, Fairfield, USA; 2 Medicine, California Institute of Behavioral Neurosciences & Psychology, Fairfield, USA

**Keywords:** takotsubo cardiomyopathy, stress cardiomyopathy, torsades de pointes (tdp), ventricular arrhythmias, cardiac arrhythmia

## Abstract

Takotsubo cardiomyopathy (TTC), also known as broken heart syndrome, stress cardiomyopathy (SCM), or apical ballooning syndrome, is a non-ischemic cardiac disease with an initial clinical presentation that is very similar to acute coronary syndrome (ACS). Ventricular arrhythmias (VAs) contribute significantly to an increase in the rates of death in patients with TTC, especially during the acute phase, in which patients with TTC are more susceptible to develop life-threatening arrhythmias (LTA) such as ventricular tachycardia (VT), ventricular fibrillation (VF), torsades de pointes (TdP), and sudden cardiac death (SCD). However, the pathophysiology of TTC and how VA occurs are still a mystery. We aim to review previous literature and discuss the possible mechanisms of VA in TTC patients.

VA usually complicates the acute phase of the disease and worsens the long-term prognosis. Alterations of repolarization (negative T wave, prolonged QTc) indicate a high risk of arrhythmic events (TdP, VT, VF, and SCD). Catecholamine effect on myocardial cells and myocardial edema can create a substrate for the development of VA. Some of the most commonly proposed mechanisms for the development of VA in patients with TTC are coronary vasospasm, myocardial stunning due to catecholamines, re-entry, and triggered activity. Further prospective studies, including a more significant number of patients, are required to understand the disease's pathophysiology better and improve LTA management in patients with TTC.

## Introduction and background

Takotsubo cardiomyopathy (TTC), also known as broken heart syndrome, stress cardiomyopathy (SCM), or apical ballooning syndrome, is a type of non-ischemic cardiac disease characterized by an acute dysfunction of the myocardial tissue primarily affecting the left ventricle. However, the right ventricle can also be affected [1]. TTC received its name from the Japanese word "takotsubo" due to the distinctive appearance of the left ventricle (LV) resembling a jar used by Japanese fishermen to capture octopuses. The initial clinical presentation of TTC is very similar to acute coronary syndrome (ACS). Similar patterns of cardiac enzymes and changes on the electrocardiograph (ECG) are almost identical between them. The only separating factor is the absence of coronary artery disease or stenosis in TTC [2]. The most striking feature of TTC is a transient abnormality of the left ventricular motion, which results in the ballooning of the LV during systole. This ventricular dysfunction is only transitory in most cases, with a return to normal LV function within a few days or weeks [3]. The latter fact led to the belief that the disease carried an excellent prognosis. However, after years of study and many reported cases, we now know TTC prognosis is worse than what we initially expected. TTC currently represents 1%-3% [4,5] of all patients and 5%-6% [6] of women with clinical suspicion of ACS. The disease is more frequent in postmenopausal women (55 years and older) than in younger females and males. TTC is usually precipitated primarily by emotional and physical stressors; many cases could also occur without any of these stressful factors being present. The pathophysiological mechanism of TTC is very complex and, until these days, remains unclear. Multiple theories are trying to explain what has been described as multifactorial [7]. During the acute phase, patients with TTC are more susceptible to cardiovascular-related complications, including congestive heart failure (HF), ventricular arrhythmias (VAs), and cardiogenic shock [8-9]. A wide variety of arrhythmias are associated with TTC; the list includes life-threatening arrhythmias (LTA) such as ventricular tachycardia (VT), ventricular fibrillation (VF), prolongation of the QT interval leading to torsades de pointes (TdP), and, in the worst-case scenario, sudden cardiac death (SCD) [10]. Life-threatening ventricular arrhythmias (LTA) can occur during the acute phase of the disease, probably due to myocardial ischemia and catecholamines on the myocardium [11]. Ventricular arrhythmias contribute significantly to TTC mortality rates; however, the underlying VA mechanisms are still not well understood. Some of these mechanisms alter repolarization [12], re-entry, and abnormal automaticity [1]. However, their role provoking VA in TTC needs to be fully determined. We plan to conduct a review article while collecting information from previously published reviews and relevant case reports to establish the relationship between TTC and VA. Moreover, we attempt to summarize the possible mechanisms leading to them and to describe the effect on the patient's long-term prognosis in TTC.

## Review

Ventricular arrhythmic events associated with TTC come from a very long time. However, clinically relevant information was limited to only a few case reports or small observational studies, resulting in underestimating VA actual burden in patients with TTC [1]. During the acute phase, patients with TTC are more susceptible to develop VA, including VT, VF, QT interval prolongation leading to TdP, and, in the worse cases, SCD [10]. Ventricular arrhythmias contribute significantly to TTC mortality rates; however, the pathophysiological mechanisms leading to the development of these LTA are not well understood. That is why it is essential to summarize the possible arrhythmogenic mechanisms in TTC.

Long QT syndrome and torsades de pointes

ECG alterations are one of the TTC hallmarks, and one of the most significant changes is a prolongation of the QT interval, which usually develops in the first two days after the initial symptoms. A QTc greater than 500 ms is a significant risk factor for VA, particularly VF and TdP [9].

Ahn et al. [13] described a 78-year-old woman presenting with syncope after psychological stress. ECG findings on admission showed QTc measuring 580 ms and a heart rate of 40 beats per minute; on day two, QTc increased to 720 ms, which led to developing TdP. She was treated with DC cardioversion and magnesium sulfate and received a dual-chamber pacemaker. After discharge three months later, her ECG was still positive for prolongation of QTc, measuring 552 ms. Sasaki et al. [14] described a 22-year-old woman presenting with chest pain and syncope. The heart rate was 50/min. Initial ECG showed QTc of 730 ms with multifocal premature ventricular contractions (PVC) and development of TdP; then she received defibrillation, and QT normalized during hospitalization. Eleven months after discharge, she consulted for palpitations and chest discomfort, and her ECG showed QTc of 690 ms. Family history was negative for cardiovascular disease, not genetically tested. El-Battrawy, Behnes, Borggrefe, and Akin [15] presented a 72-year-old woman with angina pectoris one day after septoplasty; her initial QTc was 661 ms. The genetic test found her to have a heterozygous type 1 mutation in KCNQ1. Five weeks after discharge, her QTc was 487 ms [15].

Similarities exist across all these patients in terms of ECG findings and physical exam. According to the literature, they were predominantly women after menopause, and after a typical stressful factor, either emotional or physical (septoplasty).

Ventricular tachycardia, ventricular fibrillation, and other ventricular arrhythmias 

Cakıcı, Cetin, Polat, and Su Ner described a 67-year-old female with dizziness and palpitations after emotional stress, who presented with episodes of VT on ECG. While QTc was 680 ms [16], TdP did not occur during monitoring. She received cardioversion because of hemodynamic compromise due to VT. On follow-up, her QTc was 400 ms. Demir, Babur Güler, Güler, Güneş, and Kızılırmak [17] described a 27-year-old healthy woman admitted for endoscopic sinus surgery. She developed VF during recovery from anesthesia, treated with defibrillation. No stress other than the surgical procedure was present.

Watanabe et al. described a 65-year-old man with dyspnea and no recorded stressful factors. ECG showed TdP-type VT, which was terminated by cardio-pulmonary resuscitation without defibrillation. QTc was 693 ms during the episode, and six months after discharge, it returned to the standard value [18]. Rotondi et al. studied a 65-year-old woman with syncope after emotional stress [19]. Her QTc was 671 ms on ECG. She presented an episode of VT six months prior. She was diagnosed with TTC six years earlier, also presented with VT. She also had a history of idiopathic dilated cardiomyopathy.

Wakatsuki, Asano, Mase, Kurata, and Suzuki [20] reported an 81-year-old man with a history of atrial fibrillation and end-stage renal disease on hemodialysis who was admitted for implantation of a pacemaker. Twenty-six hours after implantation, he developed QTc 539 ms with polymorphic VT, which resulted in VF requiring electrical shocks to terminate it. Ten days later, his ECG returned to normal. Caudron, Rey, and Dacher described a 34-year-old female with clinical symptoms of acute appendicitis. She developed hemodynamic collapse due to VF after induction of anesthesia requiring electrical shocks to resolve. Her ECG after the event showed reversal to baseline [21]. Sosnowska, Bąkowski, Woronowicz, and Wożakowska [11] published a case report of a 59-year-old female with sudden cardiac arrest due to VF posterior to family psychological stress. ECG record showed supraventricular extrasystoles and QTc 550 ms [11].

TTC tends to occur more frequently in postmenopausal women, especially after a recent stressful episode [11,13,16,19]; however, younger women are also at risk [14,17,21], and incidence in men continues to increase every day [18,20]. Emotional stress is arguably the most common trigger. However, physical stress such as surgical procedures, long-standing illness, septic shock, severe pain [15,17], or implantation of a pacemaker or defibrillator [20] is the usual cause. We observed prolongation of the QT in all the above patients; thus, this carries a higher chance of presenting life-threatening ventricular arrhythmias, particularly TdP and VF (Table 1).

**Table 1:** Characteristics of case reports included in this review presenting TdP, VF, and VT. QTc: Corrected QT interval; VA: ventricular arrhythmia; VF: ventricular fibrillation; VT: ventricular tachycardia; TdP: torsades de pointes; PVC: premature ventricular contractions; ECG: electrocardiogram.

Author	Age	Gender	Stressful precipitant present	Clinical presentation	ECG findings	QTc	Progression to VA
Sosnowska et al. [11]	59	Female	Emotional	Cardiac arrest	Supraventricular extrasystoles	550 ms	VF
Ahn et al. [13]	78	Female	Emotional	Syncope	T wave inversion precordial leads	580 ms	TdP
Sasaki et al. [14]	22	Female	No	Syncope, chest pain	Negative T waves II, aVL, V2-6, PVC	730 ms	TdP
El-Battrawy et al. [15]	72	Female	Nasal septoplasty	Angina pectoris	T wave inversion I, aVL, V1-6	661 ms	
Cakıcı et al. [16]	67	Female	Emotional	Dizziness, palpitations	Inverted T waves V3-6	680 ms	VT
Demir et al. [17]	27	Female	Endoscopic surgery	VF after surgery	ST elevation I, aVL, depression V3-6		VF
Watanabe et al. [18]	65	Male	No	Dyspnea	Negative T wave V1-6	693 ms	TdP-type VT
Rotondi et al. [19]	65	Female	Emotional	Syncope	Inverted T waves II, III, aVF, V1-6	671 ms	VT
Wakatsuki et al. [20]	81	Male	Pacemaker implantation	Cardiac arrest due to VF	T wave inversion V3-6	539 ms	VF
Caudron et al. [21]	34	Female	Appendicectomy	Hemodynamic collapse	VF		VF
Williford [22]	51	Female	No	Syncope, chest pain	T wave inversion V4-6		Polymorphic VT
Giusca [23]	84	Female	No	Chest pain and sudden cardiac death	T wave inversion V1-4		VF

Coronary vasospasm

TTC usually has an overall long-term good prognosis with recovery in a matter of weeks [3]. However, not the usual course, some patients can have an increased risk for recurrent VA, which is related to recurrent coronary vasospasm [22]. We encountered two studies describing patients with coronary vasospasm leading to arrhythmic complications in TTC. The first one depicted a 51-year-old female with syncope, recurrent shortness of breath and palpitations, with polymorphic VT one year after the initial presentation [22]. The second one is of an 84-year-old woman who presented with SCD secondary to VF [23]. There was no emotional or physical stress on either one of the patients, but one had a personal history of tobacco use. Recurrence of TTC, especially presenting with VA in the setting of coronary-induced vasospasm, is not frequent. Women of young age with a history of anxiety and tobacco use have a significantly increased chance of developing myocardial damage and arrhythmias, even with a negative angiography [22]. This stunning of the myocardial tissue is temporary, and approximately only 28% of patients with TTC develop coronary artery spasm after provocation [3].

Abnormal repolarization

Streitner et al. studied the relationship between abnormal repolarization and fatal arrhythmias in 73 TTC patients at a medical center and collaborated with previously published cases from other authors. They analyzed the QTc values (corrected for heart rate) and measured T waves to assess repolarization parameters. They concluded that patients with malignant arrhythmias have, in general, lower ejection fraction than the average. They observed that the QTc showed increased values from day one to day three, and patients who developed TdP had slower heart rates [24]. We also identified this in some of the presented case reports [13,14,18]. The latter finding could indicate a direct relationship between abnormal depolarization and the genesis of VA. Song et al. studied 105 patients diagnosed with TTC who presented with or without QT prolongation. They performed daily ECG measuring QTc, ST-segment elevation, and T wave inversions. They found a higher incidence of VT/VF and SCD in patients with longer QTc [25]; TdP was also more frequent. The mortality rate was 7% during follow-up. They also believed that catecholamines play an essential role due to their effect in causing calcium overload in the myocardial cells. The action potential duration will determine the amount of calcium entering the myocytes; this can be increased in patients with long QT syndrome (LQTS) whose repolarization reserve appears reduced.

Life-threatening arrhythmias, J wave, and fragmented QRS in Takotsubo cardiomyopathy

Madias et al. reported an 8% association between VA and TTC, and thus, they suggested to include TTC as one cause of acquired LQTS and a more significant risk factor for TdP [26]. Shimizu et al. evaluated the presence of J wave and fragmented QRS (fQRS) as a predictive measure of VA. They collected data of 31 patients and found the presence of J wave and fQRS only during the hyperacute phase of disease in 30% of patients; they concluded that J wave indicates a high risk of VA/cardiac death while fQRS predisposes to VF [27]. The presence of the J wave also showed high-risk mortality. They proposed ischemic electrical alterations and possible catecholamine effect as the J wave formation mechanism, while ischemic myocardial cells explain fQRS. This finding correlates with those of another study [1], in which the authors found that VF accounts for 30% of the initial rhythm needing CPR and that presence of J wave on ECG was indicative of imminent VT.

Jesel et al. [28] studied a large cohort of 214 patients, and they found a 10.7% incidence of LTA, which were associated with abnormal conduction and low ejection fractions. These patients presented with VT and TdP; all progressed to VF needing defibrillation. Most LTA occurred on the first day of admission, and 54% of cases occurred out of the hospital and was the reason for admission. During long-term follow-up, the mortality rate was 25% for the entire population and 57% for the group with LTA. Repolarization changes and high adrenergic drive can prolong the QT and cause TdP [26]. In this study, 80% of patients presented QTc values greater than 500 ms (mean of 507 ms) [28]. The incidence of LTA is about 14% of TTC patients [29], 23% mortality rate during hospitalization, and 30% during long-term follow-up for the group with LTA [29].

Common pathophysiological mechanisms of ventricular arrhythmias in Takotsubo cardiomyopathy

Re-entry is the most common VA mechanism, especially in the presence of VT [1]. Based on areas of the myocardium affected by fibrosis and scarring, which could alter the electrical conductance, although TTC is a non-ischemic disease, the coexistence of regions with different electrical conductivities and myocardial edema might create a substrate for VA via re-entry [1]. Negative gadolinium enhancement studies suggest no scar formation and point toward triggered activity mechanism instead of re-entry [30,31].

A triggered activity can occur due to microvascular damage provoking repolarization anomalies. Furthermore, early afterdepolarization-induced triggered activity combined with excessive catecholamine activity could explain VT and TdP [1]. These early afterdepolarizations could present as ECG changes (affecting the T-U waves) [26]. The appearance of negative T waves in the acute stage of the disease may represent a more extensive myocardial damage and edema. Myocardial edema, in conjunction with prolonged QTc, increases the risk of VA and SCD [32,33]. Patients presenting with cardiac arrest had a higher mortality rate (40%) during hospitalization. High levels of catecholamines could also alter the uptake of calcium into the myocardial cells causing them to display abnormal automaticity and mediate VA [1,3,25,30].

Takotsubo cardiomyopathy and anorexia nervosa as a substrate for ventricular arrhythmias

Not much information is available about this topic in the literature. Ohwada et al. described the first three cases of an association between anorexia nervosa (AN) and TTC [34]. Only one patient had confirmed TTC, but they all shared clinical, ECG, and imaging findings consistent with TTC. They presented with hypoglycemia and electrolyte disturbances. Catecholamine levels were increased in two out of three cases, probably secondary to hypoglycemia. Two more studies describing patients with AN in the setting of TTC [35,36] with similar clinical presentation except glucose levels were within the normal range for one of them [35], whereas the other one presented with hypoglycemia [36]. Prolongation of QTc and recurrent events of TdP occurred in one patient [35] and cardiogenic shock in the other [36]. Cardiac hypotrophy in AN might alter the duration of the action potential, and electrolyte anomalies may prolong the QTc contributing to VA development in these patients [35]. TTC usually occurs in postmenopausal women, and there is a possible role of estrogen deficiency in the development of complications in these patients. AN may induce hormonal changes in younger women making them more susceptible to TTC and complications such as VA [36,37].

**Figure 1:**
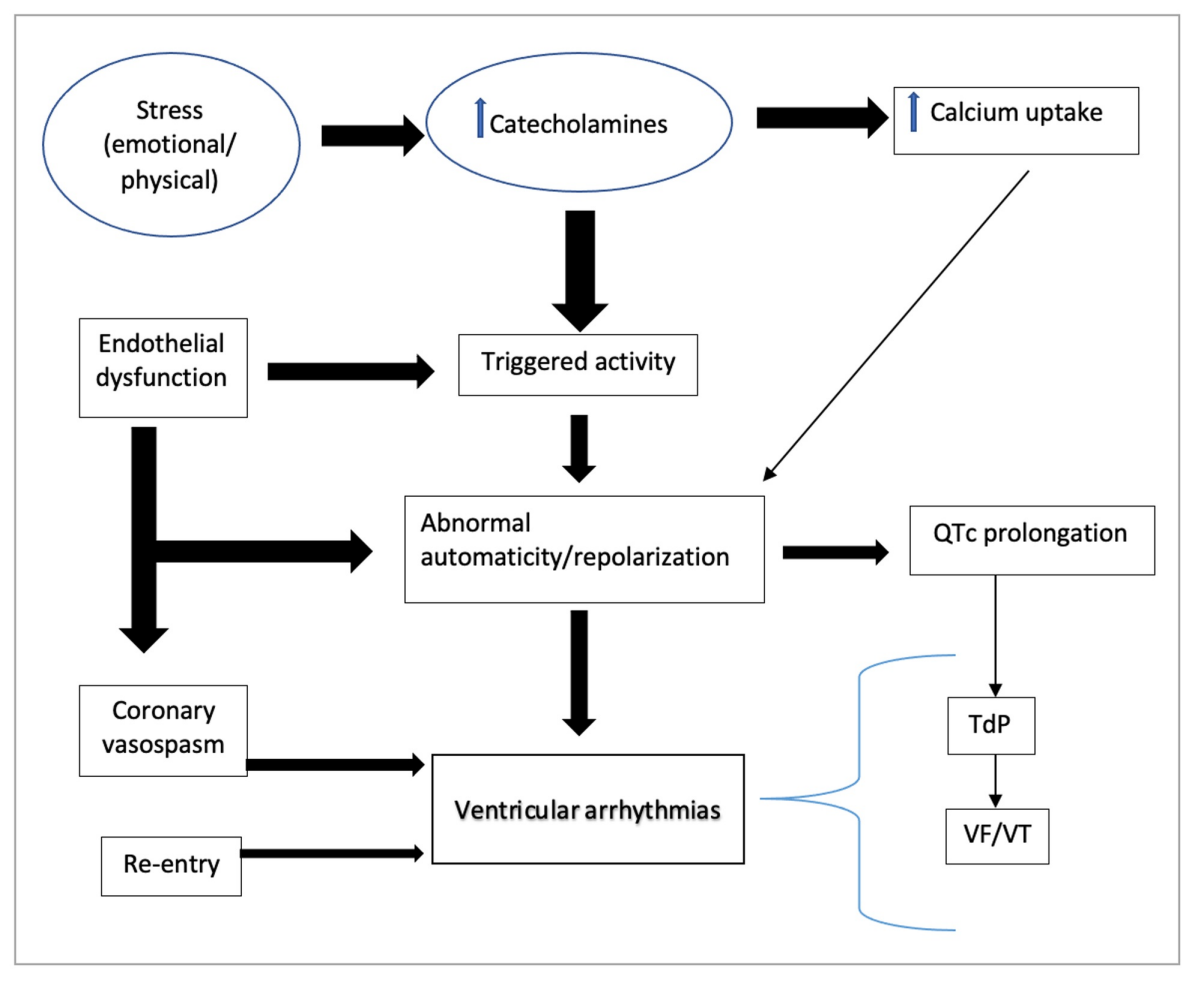
Pathogenic mechanisms of ventricular arrhythmias in takotsubo cardiomiopathy. VT: Ventricular tachycardia; VF: ventricular fibrillation; TdP: torsades de pointes; QTc: corrected QT interval.

Our review helped us summarize the most current and most accepted mechanisms that cause VA in patients with TTC (Figure 1) and has helped us understand a little more about the relationship between the clinical presentation, ECG findings, and clinical outcomes as morbidity and mortality.

Limitations

One limitation present in this review is that we only included studies from the year 2000 to present and full-text access, possibly missing important information. Another is that we did not conduct a quality assessment.

## Conclusions

Understanding the pathogenesis of TTC and its complications is a well-known challenge to clinicians around the world. In this context, VAs are present in many patients with TTC, and understanding how these two entities are related is very important. Their development's underlying pathophysiological mechanisms could help identify people at high risk of more severe cardiac events. This review aimed to summarize the most commonly proposed mechanisms provoking VA in patients affected by TTC and the effect on long-term prognosis in these patients. VA usually occurs in the acute phase of the disease and worsens the long-term prognosis of the disease. Depolarization anomalies such as T wave inversion and prolongation of QTc present in the early stage indicate a high risk of progression to severe and deadly arrhythmic events (TdP, VT, VF, and SCD). Catecholamine effect on myocardial cells and myocardial edema can create a substrate for the development of VA. Re-entry is the most common mechanism, but some studies argue it because of the lack of scar tissue in imaging studies. The latter fact points to triggered activity as one of the more accepted and probable theories; however, the exact mechanism remains uncertain.

In this review, we summarized all the possible mechanisms leading to VA and raised the awareness of a relationship between TTC and complications like VA. Also, we noticed AN could provide a substrate for the development of VA in patients with TTC, possibly due to a lack of protective effect of estrogen. Information about this topic is limited, and more studies are needed to clarify this relationship. The challenge for future scientists is to conduct more prospective studies and clinical trials assessing electrophysiological parameters in a more significant number of patients to stratify the risk and improve the prevention and management of LTA in TTC.
